# Comparing acute normovolumic hemodilution with autologous platelet-rich plasma for blood preservation during aortic surgery : study protocol for a randomized controlled clinical trial

**DOI:** 10.1186/s13063-023-07800-4

**Published:** 2023-11-18

**Authors:** Dan Zhu, Yu Li, A-yong Tian, Hong-nan Wang

**Affiliations:** Department of Anesthesiology, The First Hospital of China Medical University, 155 Nanjing North Street, Heping District, Shenyang City, Liaoning Province China

**Keywords:** Autologous platelet-rich plasma, Acute normovolumic hemodilution, Postoperative blood drainage volume

## Abstract

**Background:**

Both acute normovolumic hemodilution (ANH) and autologous platelet-rich plasma (aPRP) have been demonstrated blood-protective effects in cardiac aortic surgery; however, the efficacies of the two methods have not been compared. This study aims to compare the effects of aPRP and ANH prior to aortic surgery on postoperative bleed and other outcomes.

**Methods and analysis:**

This is a prospective, single-center, double-blind controlled clinical trial including 160 patients randomized 1:1 to receive aPRP (test group) or autologous whole blood (ANH, control group). The primary objective is to compare the drainage volumes in the two groups at 24, 48, and 72 h postoperatively. Secondary outcomes include input of allogeneic blood and blood products and durations of aortic block, extracorporeal circulation, deep hypothermic arrest of circulation, tracheal extubation, hospital stay, requirement for secondary surgical hemostasis, and application of intra-aortic balloon pump or extracorporeal membrane oxygenation in the two groups. In addition, heart rate, systolic blood pressure, diastolic blood pressure, central venous pressure, and thromboelastography recorded before blood reservation (T1), after blood reservation (T2), before blood transfusion (T3), and after the blood is returned (T4) to the transfusion will be compared between the two groups of patients.

**Discussion:**

This study will demonstrate if the use of aPRP could reduce the risk of bleeding after aortic surgery compared with ANH. The results are expected to have practical clinical applications in terms of more effective blood protection and shorter hospital stay.

**Trial registration:**

This study was registered with the Chinese Clinical Trial Registry (http://www.chictr.org.cn/) with the ID ChiCTR 1900023351.Registered on May 23, 2019.

**Trial status:**

Recruiting start date: July 1, 2019; expected recruiting end date: July 1, 2024

Version number and date: Version 2 of 05-04-2019

**Supplementary Information:**

The online version contains supplementary material available at 10.1186/s13063-023-07800-4.

## Background

Both acute normovolumic hemodilution (ANH) and autologous platelet-rich plasma (aPRP) have been demonstrated blood-protective effects in cardiac aortic surgery [1, 2]. Platelet-rich plasma (PRP) is a plasma product with a high concentration of platelets that is made by separating fresh whole blood from the patient’s body. Platelets are typically 3–5 times more concentrated in PRP than in normal whole blood, and the key components include platelets, plasma, fibrinogen, leukocytes, and other blood components. Because it is derived from autologous blood, it is rich in sources, easy to get, simple to make, safe, and effective. According to the Chinese pain expert consensus (2022 edition) on the technical specifications for the preparation of autologous platelet-rich plasma, it can produce a large number of growth factors, which play an important role in accelerating wound healing, promoting tissue regeneration and repair, and relieving pain. Absolutely, blood is a valuable resource, and blood transfusion is a vital medical procedure, and cardiac surgery is a very risky type of surgery. Surgical procedures involving the cardiac aorta are potentially dangerous, often necessitating extracorporeal circulation (cardiopulmonary bypass, CPB) or even deep hypothermia, and frequently involving a high level of intraoperative outflow [3, 4]. Blood protection and restoration of clotting function are critical steps in aortic surgery [5], and the use of homologous blood and blood products is common in patients undergoing cardiac aortic surgery. Although autologous blood recovery machines are often used for extracorporeal circulation assistance during surgery, this process washes the blood to leave concentrated red blood cells, while the platelets and plasma are wasted by washing. Although CPB preserves the platelets and plasma, the destruction and activation of red blood cells, platelet function, and coagulation factors caused by heparinization and activation of pipeline materials, mechanical rolling, and other factors can inhibit postoperative renal function and coagulation, leading to high drainage volumes and even the need for secondary open-heart hemostasis [6]. Transfusion of allogeneic platelets and plasma is beneficial in the treatment of postoperative hemorrhage. The use of platelet-rich autologous plasma in patients undergoing aortic coarctation surgery is related with a decrease in intraoperative and postoperative blood transfusions in cardiac surgery [7]. PRP usage in aortic arch replacement surgery minimizes cryopresorptive transfer, raises postoperative serum albumin and total protein levels, and lowers the frequency of pleural effusion. PRP injection had no effect on other postoperative blood parameters or CTA readings [8]. PRP is used in many aspects of blood product use in cardiothoracic surgery [9]. However, the collection and storage of these components are difficult and expensive, with numerous potential complications.

ANH is a common clinical practice for blood conservation during autologous blood transfusion and has been used in a variety of procedures. ANH has been shown to reduce the use of postoperative blood products, drainage, and the rate of secondary open-heart hemostasis in patients undergoing cardiac surgery [10]. Blood separation has been shown to have a similar effect [11]. However, there is currently a lack of randomized controlled trials, and it has been suggested that its use should be limited to aortic surgery, with further information needed regarding its use during cardiac surgery.

Although some studies have confirmed the hemoprotective effects of ANH and blood separation in aortic surgery, no randomized controlled trials have compared these two methods. We designed a study to test the hypothesis that blood separation techniques may be more effective for restoring coagulation and reducing the need for allogeneic blood products.

## Methods

### Study design {8}

This is a prospective, single-center, double-blind controlled clinical trial comparing the use of autologous platelet-rich plasma (aPRP, experimental group) and autologous whole blood (ANH, control group) in patients undergoing aortic surgery. The main study objectives include the volumes of allogeneic blood and blood products needed. We also aim to compare the durations to aortic block, extracorporeal circulation, deep hypothermic arrest of circulation, and tracheal extubation, length of hospital stay, and the incidence and mortality of secondary surgical hemostasis or application of intra-aortic balloon pump (IABP) and extracorporeal membrane oxygenation (ECMO) in both groups. Heart rate, systolic blood pressure, diastolic blood pressure, central venous pressure, and thromboelastography (TEG) were also compared before blood reservation (T1), after blood reservation (T2), before blood reserving (T3), and after (T4) blood reserving.

### Study site and patient selection

#### Patients

A total of 160 patients undergoing aortic surgery at the Department of Cardiac Surgery, First Affiliated Hospital of China Medical University, Shenyang, Liaoning Province, China, from July 1, 2019, will be recruited. All patients were randomly divided into two groups in a 1:1 ratio. The purpose and potential risks and benefits of this study will be fully explained to the patients and their families and informed consent will be obtained prior to the procedure.

#### Enrolling{16c}

The computer is responsible for assigning sequences, randomly assigning patients to groups A and B. After seeing the patient, the anesthesiologist is in charge of recruiting participants.

#### Who will take informed consent? {26a}

Informed consent is obtained from the anesthetist during the pre-operative visit or from the blinded members of the study team on the day of surgery. Written informed consent is also required from the patient’s legal representative and the investigator before any study-related procedures are started.

#### Additional consent provisions for collection and use of participant data and biological specimens {26b}

Not applicable. Collection of biological samples is not required.

#### Inclusion and exclusion criteria

The inclusion criteria are patients undergoing aortic revascularization under deep hypothermic arrest circulation in an elective or emergency setting, and patients and families provided informed consent. The exclusion criteria are a history of previous cardiac surgery, preoperative hemoglobin < 90 gL, hematocrit < 30%, platelets < 100 × 10^9^L, and cardiac insufficiency, left ventricular ejection fraction < 50%, patients who died following surgery for a variety of reasons (hemorrhagic shock, heart failure, infection, etc.), or severe aortic stenosis. The discharge criteria are good recovery of cardiac function, hemodynamic stability maintained, and blood counts and other biochemical parameters are normalized.

#### Double-blind randomization and allocation concealment

Enrolled patients undergoing aortic surgery will be divided randomly into a control group (ANH) and an aPRP group using a computer-generated sequence. Groups will be chosen before patient admission, and all patients and staff will be oblivious to their group assignment. The anesthesiologist will not be informed of the patient assignments, which are recorded and sealed in sequentially numbered opaque envelopes. Nurse anesthetists can give sealed envelopes and tell to prepare the items for the experiment based on group allocations; there are no differences in the look of the two sets of envelopes. The group assignment will be withheld from all patients and anesthesiologists. Following anesthesia induction, the anesthesiologist may perform the various surgeries in accordance with the coded number. Another anesthesiologist will document intraoperative data and followed up on the patient’s discharge. Finally, the data will be analyzed by a professional statistician.

#### Unblinding {17b}

A blinding committee is convened, which consisted of experts to oversee and guide the blinding process. At the end of the trial, after the completion of the locking library, in order to carry out the analysis, the first level of blinding, so as to know each subject as group A or B, but do not know the specific group A and B group operation. On the basis of primary blinding, statistical analysis is performed to clarify the difference between group A and group B. Secondary blinding is then performed to know the operation group information of each subject, to clarify whether it is the test group or the control group, and to further analyze the results of the clinical trial. The blinding results are recorded in a blinding form and confirmed and signed under the supervision of a blinding committee. Under the direction of a blinding committee, the outcomes of the blinding are documented in a blinding form and verified and signed.

The study will be overseen by an impartial data and safety monitoring body. The data will not be released until the final statistical analysis has been completed.

To minimize bias and avoid any influence of the surgeon on the results of the trial, all enrolled patients will be treated by the same surgical team.

## Interventions

### General anesthesia

All patients will receive general anesthesia as follows: induction with midazolam 0.05 mg/kg, etomidate 0.3 mg/kg, sufentanil 0.5 μg/kg, and cis-atracurium 0.2 mg/kg. The anesthesia machine is connected after tracheal intubation, with the tidal volume set at 6–8 mL/kg and the respiratory rate at 12 breaths/min. The intraoperative end-tidal CO_2_ pressure is maintained at 35–45 mmHg by adjusting the respiratory parameters. Anesthesia is maintained with continuous pumping of 4–8 mg/kg/h propofol, with sufentanil and cis-atracurium injections at regular intervals. After induction of anesthesia, a large-diameter catheter is placed in the patient’s right internal jugular vein. The red blood cells were reinfused back into the patient after collection, and the collected aPRP was infused back into the patient after extracorporeal circulation following neutralization of heparin with fisetin.

There is no universally accepted criterion for determining the ideal amount of whole blood to collect for ANH, which is sometimes established clinically by diluting the target hematocrit or blood volume. However, according to the 2019 expert agreement on the clinical treatment of autologous blood transfusion, the total volume for blood collection should be as follows: 10–15% of total blood volume, with an increase to 20–30% of total blood volume in rare instances or in patients with improved health status. Accordingly, autologous blood is taken from the internal jugular vein following anesthesia and central venous cannulation with a volume of 8–10 ml/kg and Hct > 30% before CPB in the ANH group, and acute isovolumic hemodilution with sodium acetate-Ringer solution is conducted. If the collected blood is required for extracorporeal circulation reasons, this test will exclude this case. Ringer’s acetate will be used as a volumetric substitute to maintain hemodynamic stability throughout the blood collection process at a ratio of 1:1.5 (volume replenishment). Previous studies used crystalloids at a ratio of 1:3, and a pre-study test demonstrated that a ratio of 1:1.5 provided safe and effective volume replacement. At the end of the procedure, the patient is returned to the cardiac surgical care unit with assisted ventilation with a respiratory ball.

A five-lead electrocardiogram, ambulatory blood pressure, oxygen saturation, end-expiratory CO_2_ pressure, near-infrared spectral cerebral oxygen saturation, and nasopharyngeal temperature will be monitored throughout the procedure and blood gas analysis and TEG will be performed regularly.

### aPRP collection

aPRP will be collected from a large-bore central venous access via gravity drainage, with approximately 15–20 mL/kg whole blood collected prior to heparin administration. The collected whole blood will be divided into aPRP and red blood cells using an autologous transfusion system (Sorin Electa Essential; Sorin, Italy). The target yield of aPRP is 10 mL/kg. The amount of PRP collected can be determined using kilogram mass, and most research [2, 11] specify a collection of 10 mL/kg of aPRP, with the exception of the 1994 study [12], which collected 15 mL/kg of aPRP.

Specific steps: An anticoagulation prefilled blood collection tube is connected to an external central venous access for blood collection. The anticoagulant solution is maintained at a constant drop to mix with the blood in the tube, and the blood entering the centrifuge chamber is centrifuged to separate aPRP and RBCs. While the blood is being drained, a crystalloid solution is infused through the peripheral vein to maintain hemodynamic stability. Blood collection was stopped when the target volume was reached. All blood collection, extraction and other operations required strict asepsis, and the resulting aPRP was stored inter-operatively at room temperature under shaking conditions. The whole separation and extraction process is completed before CPB heparinization. The aPRP will then be stored in sodium citrate collection bags at room temperature (usually 22~24 °C). When extracorporeal circulation is stopped, collected whole blood is inserted in a blood bag containing sodium citrate and transfused, heparin is neutralized with ichthyoglobulin, and no active bleeding is detected throughout operation. The anesthesiologist decided to perform aPRP.

### Plans to promote participant retention and complete follow-up {18b}

No applicable. We believe that the focus of our experiment is on various markers during the perioperative phase. On the day of the procedure, the patient will be monitored until the patient is discharged from the PACU. We shall employ the same data collecting and statistical forms for all experimental data.

### Follow-up

Patients in this trial will be followed up by an investigator once a day on postoperative days 1–3 to record the drainage volume and the administration of IABP and ECMO.

### Data collection and management

Dedicated staff will record all data and complete a case report form for each patient. Relevant demographic characteristics and the patients’ past medical histories, including age, height, weight, and diagnosis, will be collected at the time of patient inclusion. Intraoperative data, including mean arterial pressure, heart rate, oxygen saturation, and central venous pressure, will be recorded before the beginning and after the end of collection and before and after the return infusion. TEG testing will also be performed before and after the collection and before and after the return infusion. The decision to use blood products such as fibrinogen, prothrombin complex, coagulation factors, and platelets will be made based on the TEG results.

### Determination of sample size

We calculate the required sample size based on the drainage volume (mean ± standard deviation) at 24 h after the end of the manual procedure. Our preliminary study showed that the drainage volumes at 24 h postoperatively in the aPRP and control groups were 316 ± 102 and 378 ± 133 mL, respectively. Eighty patients per group were therefore needed to detect a difference with a power of 80% and a type I error rate of 5% using the PASS software.

### Subgroup analyses{20b}

At this moment, subgroup analyses have not been taken into account. The variables employed in the minimization algorithm will be subjected to subgroup analyses, but only for the main result, if necessary.

### The treatment of non-adherence and missing data{20c}

If there is a clear violation of the protocol, the trial should be stopped, and the subject’s data should be blinded and not included in the final statistical results; if it can be adjusted in time and does not make a significant difference, the data set can be retained. If there are missing data cases, replacement of the data can be tried if necessary, based on the dataset’s characteristics and qualities.

### Interim analyses {21b}

Regular meetings are held to evaluate and correct the trial as necessary. However, in this trial, interim analysis is not planned.

### Statistical analysis

Statistical analysis will be carried out using the SPSS software, version 26.0 for Windows (SPSS, Inc., Chicago, IL, USA). Continuous variables will be described as mean (standard deviation) or median (25% and 75% percentiles) and analyzed using independent *t*-tests or Mann–Whitney *U* tests, respectively. Categorical variables will be described as a frequency or percentage and analyzed by *χ*^2^ tests. Prior to statistical analysis, each continuous variable will be analyzed to determine if it is normally distributed. If the assumption of a normal distribution is violated, the appropriate transformation will be used, and analysis using repeated measures analysis of covariance (ANCOVA) models may be more effective.

### Important protocol modifications{25}

If problems are discovered and the trial protocol needs to be modified or adjusted, the reasons, contents, and timing of the modification should be stated, and the application should be submitted to the ethics committee for approval and consent before implementation; if the modification of the trial protocol during the clinical trial involves the informed consent of the subjects, the informed consent form should be changed; or if there is a new modification or addition to the trial protocol, the informed consent form should be changed.

### Confidentiality{27}

All researchers are professional clinical or scientific experts and will strictly abide by the regulations on patient information protection and ethical safety. Personal information that has been recorded is handled with the utmost confidentiality and processed and stored in line with data protection legislation. Except for parties directly involved in the subject’s care and organizations to whom the subject has given express agreement, no patient-identifiable information will be transmitted to anyone else.

### Disclosure policy

In accordance with standard protocol guidelines, the unblinded data from the trial will not be available until the primary results have been published. Blinding will not be performed until the end of the study. A clinical article reporting the primary and secondary outcomes of the study and the results will be disseminated, regardless of the size or direction of the impact. A full study report, anonymous participant-level dataset, and statistical codes for the results will be made publicly available no later than 3 years after completion of the study. The authors received no industry backing for this study.

### Patient and public involvement

Patients and/or the public were not involved in the design, conduct, reporting, or dissemination plans of this research.

## Discussion

The conservation of blood while minimizing the need for allogeneic transfusions and blood product input during cardiac aortic surgery is an important area of research. Both reserved whole blood and blood separation procedures are used to preserve the patient’s blood components, replenish their volume with crystals or colloids, and return them back to the patient as needed. Blood separation has the benefit of being more widely usable; concentrated red blood cells can be given back to the patient, especially patients with unstable hemodynamics, while only platelet-rich plasma is kept. Blood separation is currently usually used intraoperatively, whereas whole blood reserve can be started both preoperatively and intraoperatively. Platelet and plasma mono-collection have long been used in other clinical areas [13], and the recent miniaturization and popularization of equipment has further promoted the use of autologous blood separation and return transfusion techniques. Given the complexity of the coagulation system, the widespread use of TEG has provided clinical anesthesiologists with a useful tool to guide coagulation recovery [14]. TEG was performed at various time points in the current study to help standardize patient coagulation management.

Both methods of blood conservation involve volume therapy during blood collection, and previous studies suggest that a 1:3 ratio is required for rehydration [15], especially for simple crystalloids. Our preliminary experiments indicated that a ratio of 1:1.5 was sufficient. However, we need to be aware of the occurrence of hypocalcemia, and we therefore routinely added supplemental calcium in our experiments.

Our preliminary experiments also demonstrated the need for the precise addition of citrate to ensure the efficient collection of whole blood and PRP. We regulated the factors likely to affect the amounts of whole blood and PRP collected by using an infusion pump and a 1:10 ratio. We were unable to quantify platelet numbers and quality reliably due to platelet preservation, which may result in some data bias and inaccuracy.

Postoperative coagulation recovery is vital [16], and the surgeon’s hemostatic abilities are critical for reducing the risk of postoperative drainage and secondary open-heart hemostasis. However, a skilled team is needed to complement the surgeon’s performance.

ANH is easier to conduct and presents fewer challenges than blood separation, which necessitates the use of specialized equipment and materials as well as the training of experts. However, blood separation procedures are more controllable, the concentrated red blood cells can be easily returned for transfusion, and the hemodynamics are more manageable. Because of the short in vitro anticoagulation preservation time, whole blood preservation is less mechanically stimulating than blood separation, but platelets and red blood cells maintained together may affect platelet function. However, some platelet activation is unavoidable during blood separation, and no studies have yet addressed this issue. Notably, there is currently no perfect solution, and further studies are needed to determine the optimal methods for blood conservation.

### Supplementary Information

**Additional file 1.**

## Data Availability

Data and materials are available on reasonable request. Additional data may be made available upon request.

## Abbreviations

ANH: Acute normovolumic hemodilution
APRP: Autologous platelet-rich plasma
